# Caso de hipocalcemia grave secundaria a síndrome de Fahr

**DOI:** 10.23938/ASSN.1138

**Published:** 2026-02-18

**Authors:** Miren Vallejo Ruiz, Inés Moral Presa

**Affiliations:** Servicio Navarro de Salud-Osasunbidea Hospital Universitario de Navarra Servicio de Análisis Clínicos Pamplona España

**Keywords:** Hipocalcemia, Hipoparatiroidismo, Calcificaciones cerebrales, Enfermedades Raras, Síndrome de Fahr, Hypocalcemia, Hypoparathyroidism, Brain calcifications, Fahr´s Syndrome, Rare Diseases

## Abstract

El síndrome de Fahr es una enfermedad rara caracterizada por calcificaciones cerebrales bilaterales, generalmente asociadas a alteraciones del metabolismo fosfocálcico, siendo el hipoparatiroidismo idiopático la causa más frecuente.

Se presenta el caso de un varón de 56 años con convulsiones, alteración del comportamiento con hipocalcemia severa, hiperfosfatemia y niveles bajos de PTH. Las pruebas de imagen evidenciaron las calcificaciones típicas de este síndrome, siendo la tomografía computarizada la prueba de imagen de referencia. El diagnóstico se apoya en la combinación de hallazgos clínicos, analíticos y radiológicos, y es crucial descartar otras causas de calcificaciones cerebrales como enfermedades autoinmunes, infecciosas o genéticas (enfermedad de Fahr). El tratamiento consiste en corregir la hipocalcemia y mantener una monitorización estricta del calcio.

Un diagnóstico precoz del hipoparatiroidismo puede prevenir las complicaciones neurológicas del síndrome de Fahr, siendo esencial el papel del laboratorio en la identificación de esta patología y su adecuado manejo.

## Abstract (en)

Fahr’s syndrome is a rare neurological disease characterized by bilateral intracranial calcifications associated with disturbances in calcium-phosphate metabolism, most commonly due to hypoparathyroidism.

We report a 56-year-old man presenting with generalized seizures and behavioral changes. Laboratory evaluation revealed severe hypocalcemia, hyperphosphatemia, and low parathyroid hormone levels. Brain computed tomography demonstrated symmetrical calcifications involving the basal ganglia and other subcortical structures, consistent with Fahr’s syndrome. Diagnosis was established through the integration of clinical features, biochemical abnormalities, and neurological findings, after excluding alternative causes of intracranial calcifications, including infectious, autoimmune, and genetic conditions. Management focused on prompt correction of hypocalcemia and long-term metabolic control with close biochemical monitoring.

Early recognition of hypoparathyroidism is essential to prevent neurological complications and improve outcomes. This case highlights the pivotal role of laboratory assessment in the diagnosis and management of Fahr’s syndrome.

## INTRODUCCIÓN

El Síndrome de Fahr es una enfermedad neuro-mineral caracterizada por la presencia de calcificaciones intracraneales simétricas, de etiología idiopática, que afectan predominantemente a ganglios basales, cerebelo y sustancia blanca periventricular^1^. Su prevalencia es inferior a un caso por millón de habitantes y presenta una clínica heterogénea que incluye alteraciones cognitivas, trastornos del comportamiento y síntomas extrapiramidales^1^. Entre las causas secundarias más frecuentes están las alteraciones del metabolismo fosfocálcico, siendo el hipoparatiroidismo la más común^1^.

El síndrome de Fahr constituye una causa infradiagnosticada de síntomas neurológicos graves, por lo que se presenta este caso con el objetivo de fomentar la sospecha clínica de esta entidad y promover el diagnóstico precoz del hipoparatiroidismo, a fin de prevenir complicaciones neurológicas irreversibles.

## CASO CLÍNICO

Varón de 56 años, sin antecedentes personales de interés, que acudió al servicio de urgencias por presentar un episodio comicial tónico-clónico generalizado, con alteración del nivel de consciencia y traumatismo craneoencefálico asociado. El paciente refirió, además, calambres musculares frecuentes y alteración del comportamiento de tipo obsesivo desde hace aproximadamente un año. Como antecedentes familiares destaca un hermano con diabetes insípida central.

En la analítica sanguínea solicitada al laboratorio de urgencias se observó un marcado descenso del calcio sérico total (5,4 mg/dL) con un calcio corregido por albúmina de 5,3 mg/dL, hiperfosfatemia (8 mg/dL) y elevación moderada de creatinina (1,48 mg/dL), con un filtrado glomerular disminuido (52 mL/min/1,73 m^2^). Además, presentaba discreta leucocitosis sin desviación izquierda y leve anemización, sin presencia de acidosis ni hiperlactacidemia en la gasometría venosa. El sedimento urinario fue negativo para tóxicos y con iones en rangos normales. Al tratarse de un valor crítico de calcio sérico, el laboratorio lo notificó inmediatamente al facultativo solicitante quien, ante la sospecha de tetania por hipocalcemia, indicó ingreso hospitalario para reposición iónica con gluconato cálcico, así como nuevos controles analíticos.

Durante el ingreso se amplió el estudio analítico; la disminución de la hormona paratiroidea o parathormona (8 pg/mL), un leve déficit de vitamina D (19 ng/mL) y la calciuria ligeramente disminuida (90 mg/24 h, rango normal: 100-300) fueron los hallazgos analíticos más relevantes (Tabla 1).

**Tabla 1:** Parámetros analíticos más relevantes en distintos momentos del seguimiento

Parámetro (unidades)	Valores de referencia	Urgencias	Ingreso día +1	Ingreso día +2	Revisión +2 meses
Glucosa (mg/dL)	74-100	87	86	87	98
Urea (mg/dL)	17-43	51	46	37	49
Creatinina (mg/dL)	0,81-1,44	1,48	1,44	1,22	1,55
Filtrado glomerular* (mL/min/1,73 m^2^)	>60	52	54	66	49
Albumina (g/L)	35-52	45	36	38	46
AST (UI/L)	5-50	51	-	53	20
ALT (UI/L)	5-50	24	22	22	-
Sodio (mmol/L)	136-146	139	138	141	140
Potasio (mmol/L)	3,5-5,1	3,9	4	3,9	4,2
Cloruro (mmol/L)	101-110	96	103	102	99
Calcio (mg/dL)	8,4-10,2	5,4	5,5	6,6	8,5
Calcio corregido por albumina (mg/dL)	8,4-10,2	5,3	6,1	6.8	8.0
Fosfato (mg/dL)	2,5-4,5	8	6	7,2	5
Magnesio (mg/dL)	1,8-2,6	1,8	1,7	1,7	1,8
PTH (pg/mL)	14-80	-	8	8	12
Vitamina D (ng/mL)	>20 (suficiencia)	-	19	16	-
TSH (mU/L)	0,38-5,33	-	0,31	0,25	-
T4L (ng/dL)	0,61-1,12	-	1,2	1,2	-

* CKD-EPI; AST: aspartato aminotransferasa; ALT: alanina aminotransferasa; PTH: hormona paratiroidea; TSH: Tirotropina; T4L: tiroxina libre.

Desde el laboratorio se amplió el perfil férrico, poniendo de manifiesto una anemia ferropénica con niveles normales de folato, cobalamina, vitamina E y retinol. El estudio tiroideo sugirió la presencia del síndrome del enfermo eutiroideo, con anticuerpos antitiroideos negativos. También se solicitaron estudio de autoinmunidad, incluyendo anticuerpos contra los canales de calcio, y serologías, con resultados negativos. Se estableció el diagnóstico de enfermedad renal crónica (ERC) estadio IIIA. 

Las pruebas de imagen realizadas fueron una tomografía axial computarizada (TAC) que manifestó calcificaciones bilaterales en ganglios basales, cerebelo y sustancia blanca, y una resonancia magnética nuclear (RMN) que mostró una hiperseñal en los ganglios basales y los núcleos dentados del cerebelo, con caída de señal en la secuencia SWI (*Susceptibility-Weighted Imaging*), en relación con las calcificaciones observadas en la TAC. De acuerdo a estos hallazgos, se diagnostica al paciente de hipoparatiroidismo primario idiopático y, junto con los estudios complementarios, de síndrome de Fahr probablemente secundario a esta condición.

Durante la hospitalización se inició perfusión de calcio y calcitriol, observando una mejoría clínica y bioquímica (calcemia corregida de 6,1 mg/dL). Una vez que el paciente alcanzó la estabilidad hemodinámica y renal, se procedió a su alta hospitalaria con seguimiento ambulatorio, tratamiento sustitutivo y recomendaciones dietéticas (evitar productos que contengan fósforo y fármacos como fenitoína y valproato que interfieran en la absorción de calcio y fosfato).

**Tabla 2:** Línea cronológica de eventos clínicos relevantes durante el seguimiento del paciente

Día	Evento relevante
0	Crisis convulsiva
	Hipocalcemia grave detectada
	Ingreso hospitalario
1	Inicio de reposición iónica con gluconato cálcico IV
	Evaluación completa (hormona paratiroidea, vitamina D, etc)
2-3	Diagnóstico de hipoparatiroidismo
	Hallazgos compatibles con síndrome de Fahr en neuroimagen
4	Inicio de tratamiento con calcitriol
	Mejoría progresiva
7	Alta hospitalaria
	Seguimiento endocrino y neurológico
60	Calcemia normalizada
	Sin síntomas Revisión ambulatoria

En la revisión a los dos meses, el paciente refirió remisión completa de los síntomas, en consonancia con la evolución favorable de los parámetros analíticos: mejoría progresiva de los niveles de calcio (8,5 mg/dL) y de calcio corregido por albúmina (8,0 mg/dL), con descenso del fósforo a valores próximos al rango de normalidad (5 mg/dL). Aunque la PTH mostraba una mejoría respecto a determinaciones previas, continuaba por debajo del rango normal (12 pg/mL), lo que justificó el mantenimiento del tratamiento sustitutivo. La Tabla 2 presenta la línea temporal de los principales eventos clínicos.

## DISCUSIÓN

El caso descrito ilustra cómo el reconocimiento precoz de una alteración del metabolismo fosfocálcico, como la hipocalcemia, puede conducir al diagnóstico de una entidad infrecuente como el síndrome de Fahr, permitiendo prevenir secuelas neurológicas potencialmente irreversibles.

La presencia de calcificaciones cerebrales simétricas en ganglios basales y otras estructuras, asociadas a síntomas neuropsiquiátricos, define el espectro de enfermedades conocidas como enfermedad de Fahr o síndrome de Fahr, según su etiología sea primaria o secundaria, respectivamente^2^. Ambas sustentan su etiopatogenia en una disfunción de la barrera hematoencefálica, lo que favorece la precipitación de minerales dentro del tejido cerebral, principalmente calcio^3^.

El síndrome de Fahr suele manifestarse entre los 30 y 60 años, sin distinción por sexo^4^. Tiene una presentación clínica inespecífica, que explica el habitual retraso en su diagnóstico, predominando manifestaciones neuropsiquiátricas como deterioro cognitivo progresivo y alteraciones de la marcha^1^. Nuestro paciente presentaba desde hace un año múltiples calambres musculares y alteraciones psiquiátricas de tipo obsesivo, que precedieron al episodio que motivó el estudio. Aunque la mayoría de los pacientes presentan síntomas de hipocalcemia, hasta un 20% son clínicamente asintomáticos y diagnosticados incidentalmente en exámenes radiológicos^1^. La gravedad de la sintomatología está relacionada con el grado de hipocalcemia y la rapidez con la que se instaura^5^. Un diagnóstico correcto del síndrome de Fahr requiere confirmar la hipocalcemia verdadera en función de los niveles de albumina sérica, y evaluar el calcio iónico en relación a las alteraciones del equilibrio ácido-base^5^.

Dentro de las causas metabólicas de calcificaciones intracraneales, las alteraciones del metabolismo de la PTH, especialmente el hipoparatiroidismo, es la más frecuente. En una revisión publicada de 150 casos, el hipoparatiroidismo representó el 23,3% de los síndromes de Fahr, y el hipoparatiroidismo secundario un 15,3%^1^. Este trastorno endocrino es causado por una deficiencia o ausencia de secreción de parathormona por las glándulas paratiroides. Esta hormona es esencial para regular el calcio y el fósforo en el organismo, controlando la actividad ósea y la reabsorción renal de estos minerales^6^. El hipoparatiroidismo puede ser primario (debido a daño directo o quirúrgico de las glándulas), secundario a otras enfermedades (enfermedad renal crónica, o deficiencia grave de magnesio) o idiopático/familiar^6^. En una serie publicada de 223 pacientes diagnosticados de síndrome de Fahr secundario a hipoparatiroidismo, se concluye que el hipoparatiroidismo idiopático/familiar es la causa más frecuente del cuadro, responsable de un 39% de los casos, seguido del posquirúrgico (35,4%) y del pseudohipoparatiroidismo (25,6%), caracterizado por presentar resistencia periférica a niveles normales de hormona paratiroidea^7^. Excepcionalmente se ha descrito al hiperparatiroidismo como causa de este síndrome^8^.

El diagnóstico definitivo del síndrome de Fahr se basa en la asociación de hipocalcemia, hiperfosfatemia, hipocalciuria y disminución de los niveles séricos de hormona paratiroidea, tal y como ocurre en el paciente de nuestro caso.

**Figura 1:**
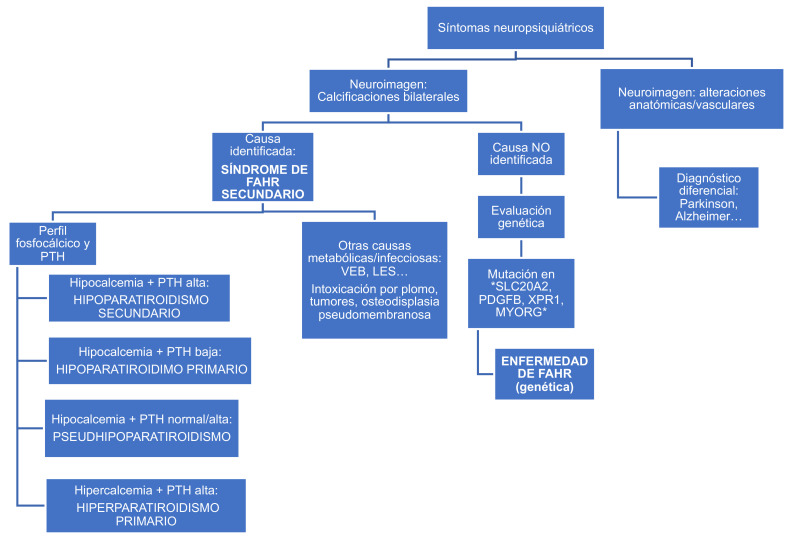
Algoritmo diagnóstico ante hallazgo de calcificaciones cerebrales y sintomatología neuropsiquiátrica.

PTH: hormona paratiroidea; VEB: virus de Epstein-Barr; LES: lupus eritematoso sistémico; SLC20A2, PDGFRB, XPR1, MYORG: genes implicados en la enfermedad de Fahr.

Se propone un algoritmo clínico para el abordaje diagnóstico en pacientes con síntomas neuropsiquiátricos, diferenciando entre causas metabólicas (síndrome de Fahr secundario) y genéticas (enfermedad de Fahr). El perfil fosfocálcico y los niveles de PTH permiten orientar hacia las principales alteraciones endocrinas. La hipocalcemia constituye el estímulo más potente para la secreción de hormona paratiroidea, por lo que su determinación resulta fundamental para el diagnóstico: si su concentración es baja en contexto de hipocalcemia, orienta hacia hipoparatiroidismo. La elevación de fosfato apoya el diagnóstico, mientras que cifras bajas sugieren hiperparatiroidismo secundario. En ausencia de una causa identificable, se recomienda realizar una evaluación genética (Fig. 1).

El TAC cerebral es la prueba de elección para la identificación de calcificaciones intracerebrales, siendo característico el patrón bilateral y simétrico en los ganglios basales, núcleos dentados, cerebelo y sustancia blanca cerebral^9^. La RMN suele mostrar señales hiperintensas en imágenes ponderadas en T1 y T2 en estas áreas^1^^,^^10^. Es necesario excluir otras causas de calcificaciones intracerebrales como enfermedades sistémicas, autoinmunes como lupus eritematoso sistémico (LES), enfermedad celíaca, quimioterapia, e infecciones víricas por el virus de la inmunodeficiencia humana (VIH) o el virus de Epstein-Barr (VEB), entre otras^9^. En estos casos, las calcificaciones suelen ser asimétricas y extraganglionares, a diferencia de las características del síndrome de Fahr^11^.

En ausencia de alteraciones metabólicas, debe realizarse el diagnóstico diferencial de la enfermedad de Fahr, de origen genético con un patrón de herencia autosómico dominante y penetrancia incompleta^11^. Recientemente se ha identificado el locus primario en el brazo largo del cromosoma 14 (14q9)^12^. Se han descrito variantes genéticas en los genes SLC20A2, XPR1, PDGFRB y PDGFB, aunque en un 50% de los casos no se logra identificar la alteración^13^. El paciente de este caso clínico no es subsidiario de evaluación genética por tratarse de un síndrome de Fahr secundario a hipoparatiroidismo primario de origen idiopático. Ante la sospecha de enfermedad de Fahr podemos apoyarnos en los criterios de diagnóstico adaptados de Moskowitz y col^2^ y Ellie y col^14^: disfunción neurológica progresiva; evidencia radiográfica de calcificación bilateral en ganglios basales y otras regiones cerebrales; ausencia de anomalías bioquímicas que sugieran endocrinopatías, trastornos mitocondriales u otros trastornos sistémicos; ausencia de infecciones, tóxicos o traumatismos; y antecedentes familiares con herencia autosómica dominante.

El pronóstico de la enfermedad es variable con tendencia neurodegenerativa^15^. El objetivo es mantener la calcemia en rango para evitar complicaciones como hipercalciuria, nefrolitiasis y nefrocalcinosis, lo que requiere una estrecha monitorización de la calcemia^16^. El pilar del tratamiento es el uso de calcio y vitamina D activa (calcitriol)^1^^,^^5^, que en el caso presentado logró una clara mejoría clínica del paciente y la normalización de la calcemia a los dos meses del diagnóstico.

En conclusión, el SF es una entidad poco frecuente que debe considerarse en el diagnóstico diferencial de pacientes con hipocalcemia y manifestaciones neurológicas o psiquiátricas. El paciente presentado mostraba una hipocalcemia grave, asociada a hiperfosfatemia, hormona paratiroidea baja y neuroimagen con calcificaciones cerebrales bilaterales, compatible con un hipoparatiroidismo primario. El laboratorio desempeña un papel crucial tanto en el diagnóstico como en la monitorización, permitiendo una intervención terapéutica precoz que mejora significativamente el pronóstico del paciente al prevenir las complicaciones derivadas de dicha calcificación cerebral y optimizando el manejo a largo plazo.

## Data Availability

Se encuentran disponibles bajo petición al autor de correspondencia.
